# Osteopathic structural exam clinical finding in diagnoses of infant positional plagiocephaly and torticollis

**DOI:** 10.51894/001c.166032

**Published:** 2026-08-01

**Authors:** Bailey Walters, Nicole Wong, Angela S. Lee, John M. Popovich Jr., Angelina Iafano

**Affiliations:** 1 College of Osteopathic Medicine Michigan State University

## 129

### Introduction

Positional Plagiocephaly (PP), unilateral head flattening in infants, has been reported in up to 46.6% of infants by 7-12 weeks of age. Left untreated, PP can lead to craniofacial asymmetry and delays in gross motor development. Untreated torticollis, ipsilateral head tilt and contralateral rotation likely secondary to sternocleidomastoid hypertonicity, is a recognized risk factor for PP and cervical spine deformity. Although treating PP/torticollis with osteopathic manipulative treatment (OMT) has been reported, documentation of diagnosed somatic dysfunction (SD) in infants with plagiocephaly and/or torticollis is limited. Hence, this case report characterizes SD in two infants with PP and torticollis from initiating OMT until discharge.

### Case description

Two female patients (both age 2 months) presented to the MSU Osteopathic Manipulative Medicine Clinic with right posterior head flattening and right head turn preference. At the initial visit, each patient underwent a full body osteopathic structural examination. Both patients were subsequently diagnosed with PP and torticollis, with initial clinical finding of cranial vault asymmetry 10mm and 7mm for Patient1 and Patient2, respectively. Both patients also demonstrated left condylar compression, left clavicle restriction, right sacroiliac restriction, and right pelvis restriction, which correlated with their right turn preference and right-sided posterior PP. Additionally, SD was identified in the thoracic, rib, and lumbar regions. SD was treated with OMT by the same osteopathic physician. Across five OMT sessions, Patient1 had a 94.74% decrease in SD (from 19, 16, 16, 8, to 1 positive findings over 4 months), whereas Patient2 had a 62.5% decrease (from 16, 15, 13, 14, to 6 positive findings over 3 months). No side effects were noted after OMT. Both patients were discharged at visit 5, as torticollis was resolved and PP improved.

### Discussion

OMT, a non-invasive, non-pharmacologic intervention, was well tolerated and demonstrated SD reductions and clinical improvements in infants with PP and torticollis. Identifying SD patterns in this patient population may result in more efficient osteopathic assessment and effective treatment strategies. Although the findings from this case report are not entirely generalizable, the observations will inform future studies aimed at identifying persistent SD patterns in patients with PP and torticollis.

